# Anatomy of superior olivary complex and lateral lemniscus in Etruscan shrew

**DOI:** 10.1038/s41598-024-65451-0

**Published:** 2024-06-26

**Authors:** Alina C. Zacher, Felix Felmy

**Affiliations:** 1 Institute of Zoology, University of Veterinary Medicine Foundation, Buenteweg 17, 30559 Hannover, Germany; 2 Hannover Graduate School for Neurosciences, Infection Medicine and Veterinary Sciences (HGNI), Buenteweg 2, 30559 Hannover, Germany

**Keywords:** Auditory brainstem, Etruscan shrew, Inferior colliculus, Lateral lemniscus, Superior olivary complex, Auditory system, Neural circuits

## Abstract

Based on the auditory periphery and the small head size, Etruscan shrews (*Suncus etruscus*) approximate ancestral mammalian conditions. The auditory brainstem in this insectivore has not been investigated. Using labelling techniques, we assessed the structures of their superior olivary complex (SOC) and the nuclei of the lateral lemniscus (NLL). There, we identified the position of the major nuclei, their input pattern, transmitter content, expression of calcium binding proteins (CaBPs) and two voltage-gated ion channels. The most prominent SOC structures were the medial nucleus of the trapezoid body (MNTB), the lateral nucleus of the trapezoid body (LNTB), the lateral superior olive (LSO) and the superior paraolivary nucleus (SPN). In the NLL, the ventral (VNLL), a specific ventrolateral VNLL (VNLLvl) cell population, the intermediate (INLL) and dorsal (DNLL) nucleus, as well as the inferior colliculus’s central aspect were discerned. INLL and VNLL were clearly separated by the differential distribution of various marker proteins. Most labelled proteins showed expression patterns comparable to rodents. However, SPN neurons were glycinergic and not GABAergic and the overall CaBPs expression was low. Next to the characterisation of the Etruscan shrew’s auditory brainstem, our work identifies conserved nuclei and indicates variable structures in a species that approximates ancestral conditions.

## Introduction

The Etruscan shrew (*Suncus etruscus*) is probably the smallest recent terrestrial mammal. This about 2 g and 4 cm large *Eulipotyphla* lives in the southern Palaearctic belt^1,2^. Due to their small body size and thus, a large surface-to-volume ratio, Etruscan shrews invest much time in hunting to meet their high-energy demands^3^. At close proximity to their prey, they make use of their sensitive vibrissae system^3,4^. In distance to a prey, they likely use their hearing abilities. Their good hearing performance may be derived from their large pinna, their estimated impedance at the eardrum and their hunting behaviour in darkness^5^. Etruscan shrews are considered to be high-frequency listeners because of their middle ear formation^5^ and their head size. Mammals with very small heads are proposed to unlikely detect sound sources based on low frequencies^6,7^. Together, these anatomical constraints might suggest that Etruscan shrews approximate the mammalian ancestral auditory periphery.

Auditory information is processed in the brainstem and hindbrain in various gross structures including the superior olivary complex (SOC), the nuclei of the lateral lemniscus (NLL) and the inferior colliculus (IC). The nuclei of these regions are considered to be functionally conserved throughout mammalian evolution^6,7^. Despite the conservative aspect, the shape and location of auditory nuclei can differ between species, especially in the SOC^6,7^ and the lateral lemniscus (LL)^8^. Following from their conserved presence, the connectivity between auditory nuclei is considered to be conserved on the gross scale. However, small but significant differences in the connectivity pattern and input location exist, as exemplified for inhibitory inputs in the medial superior olive (MSO)^9^. Therefore, the structural arrangement and the synaptic location is species-dependent.

The SOC is composed of at least six different sub-nuclei localised from medial to lateral: the medial nucleus of the trapezoid body (MNTB), the ventral nucleus of the trapezoid body (VNTB), the MSO, the superior paraolivary nucleus (SPN), the lateral superior olive (LSO) and the lateral nucleus of the trapezoid body (LNTB). Functionally, the MNTB is a temporally precise hub providing glycinergic feed-forward inhibition to SOC, NLL and thalamic nuclei^10^. Other local and long range inhibitory projections arise from the VNTB and LNTB^11–14^. The MSO and LSO are the binaural integration centres for low- and high-frequency sound source localisation^6,7^. The SPN is implicated in the detection of stimulus gaps and endings by generating offset responses^15–17^. Next to these main SOC nuclei, other less structured neuronal populations such as periolivary subregions as well as the medial and lateral olivocochlear bundle cells exist that provide important feedback to the cochlea.

Within the fibres of the LL, three nuclei can be identified termed according to their location in ventral, intermediate and dorsal nucleus of the lateral lemniscus (VNLL, INLL and DNLL)^18,19^. Within the VNLL, a subpopulation of globular neurons receive a large, somatic endbulb synapse^20,21^. Because this cell population locates at different positions within the VNLL across mammalian species, it obtained additional annotations^8,19,22^. The VNLL generates a rapid glycinergic and γ-aminobutyric-acid (GABA)-ergic feed-forward inhibition^23^ that serves to at least suppress spurious frequencies that occur during spectral splatter^24^. The INLL was indicated in cross-frequency integration in bats^25^. The reciprocal connection of GABAergic neurons in the DNLL serves various binaural functions^26–28^. Other neuronal populations in the lateral lemniscus are functionally less understood and consist of the subiculum, H-cells of the semilunar nucleus and the paralemniscal zone^29,30^. Most of the information of the SOC and the NLL is fed into the central nucleus of the inferior colliculus (ICc) in the midbrain, a main auditory integration centre.

Temporal precision is a hallmark of the auditory brainstem produced by structural and molecular adaptations. Large somatic synapses like the calyx of Held and endbulb synapses in the LNTB and VNLL allow for rapid synaptic information transfer^21,31,32^. Potassium voltage-gated channel subfamily A member 1 (Kv1.1) channels have low voltage activation accelerating sub-threshold integration to produce temporally precise supra-threshold output^33–35^. Hyperpolarization-activated cyclic nucleotide-gated channel 1 (HCN1) channels underlie hyperpolarising activated cation currents producing short integration times and offset firing^15,16,36,37^. Finally, oligodendrocytes myelinate axons and axon/ myelin thickness is adapted to specific circuit requirements^38–40^ adjusting propagation speeds. Therefore, specific structural components and molecular compositions endow the auditory system with its temporal precision and rapid voltage signalling.

Here, we determined the architecture of the SOC and the NLL in the Etruscan shrew. To identify the nuclei of these auditory regions, we labelled structural markers (microtubule-associated protein 2 [MAP2] and neuronal nuclear antigen [NeuN]), synaptic markers (vesicular glutamate transporter 1 [VGluT1] and vesicular γ-Aminobutyric acid transporter [VGAT]), transmitters (GABA and glycine), calcium binding proteins (CaBPs; calbindin [CB], calretinin [CR] and parvalbumin [PV]) and voltage-gated ion channels (HCN1 and Kv1.1). Most major nuclei of the SOC and LL could be identified and morphologically characterised, but the MSO and VNTB could not be unambiguously addressed. In addition, the expression pattern of CaBPs and voltage-gated ion channels allows suggestions about functional aspects.

## Results

**Figure 1:**
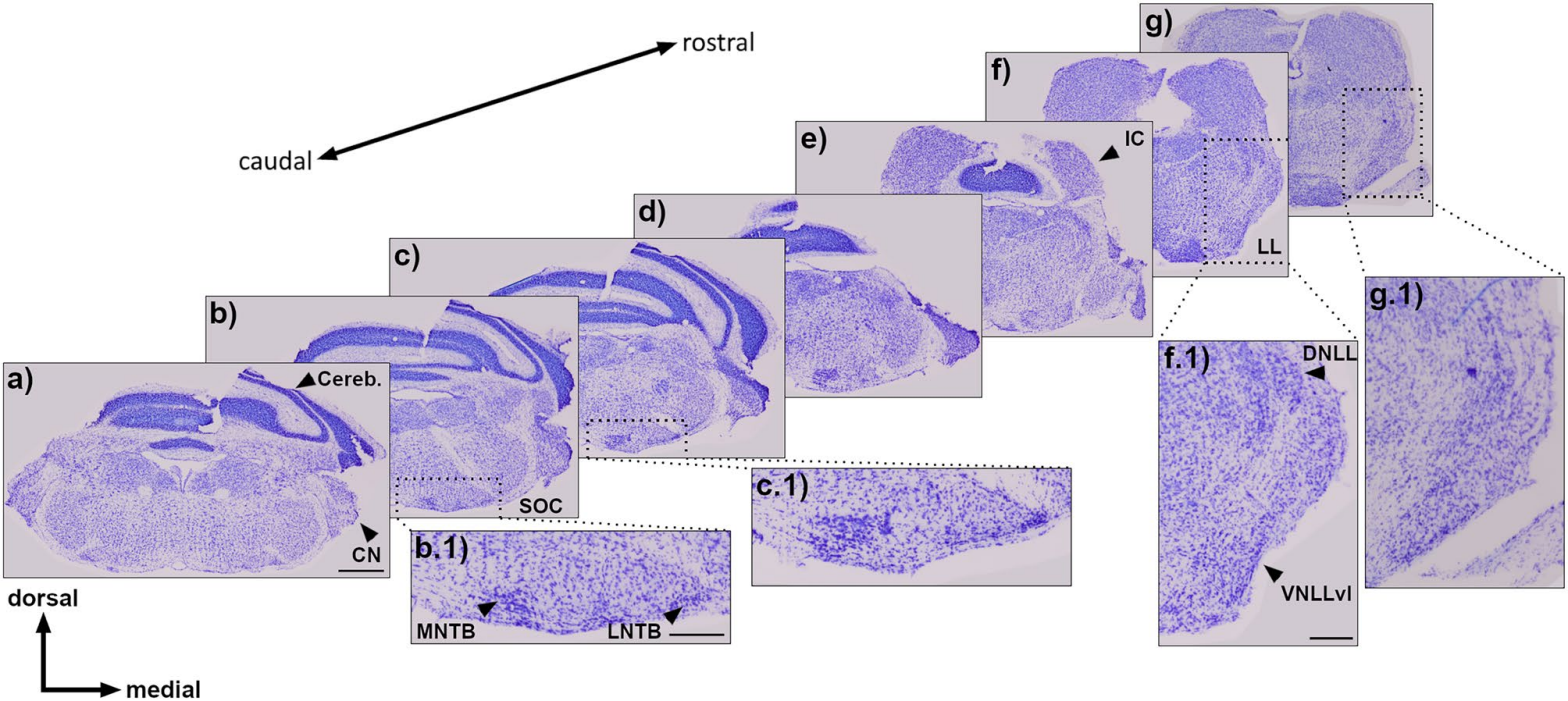
Nissl staining of brainstem to hindbrain sections. (**a**–**g**) Series of sections with every 120th µm shown from caudal to rostral, from SOC to NLL. Magnifications in (b.1) and (c.1) of the SOC are demarcated by dotted boxes, (f.1) and (g.1) show the NLL. Black arrow heads show key structures in the brain. Scale bar equals 500 µm. (b.1, c.1**)** Magnifications show SOC from two sections. Black arrow heads show key structures within the SOC. Scale bar equals 200 µm. Abbreviations: lateral nucleus of the trapezoid body, LNTB; medial nucleus of the trapezoid body, MNTB. (f.1, g.1**)** Magnifications show LL from two sections. Arrow heads show key structures of the LL. Scale bar equals 200 µm. *DNLL* dorsal nucleus of the lateral lemniscus, *VNLLvl* ventrolateral part of the ventral nucleus of the trapezoid body, *Cereb* cerebellum, *CN* cochlea nucleus, *IC* inferior colliculus, *LL* lateral lemniscus, *SOC* superior olivary complex.

In Nissl stained serial sections of the Etruscan shrew (Fig. 1a**–**g), within the SOC, the MNTB and LNTB were identified by their densely packed roundish somata (Fig. 1b.1). Other SOC nuclei like the LSO, MSO or SPN were not unambiguously delineated. On the lateral edges of the caudal hindbrain sections, the cochlea nucleus and dorsally the cerebellum (Cereb.) could be identified. Within the LL, a region of densely packed roundish somata defined the ventrolateral part of the VNLL (VNLLvl, Fig. 1f.1). The remaining VNLL was not clearly separable from the dorsally located INLL. The DNLL appeared densely packed and oval. The IC could be distinguished morphologically by its position on the dorsolateral edge of the hindbrain.

### Distribution of structural and synaptic markers

**Figure 2:**
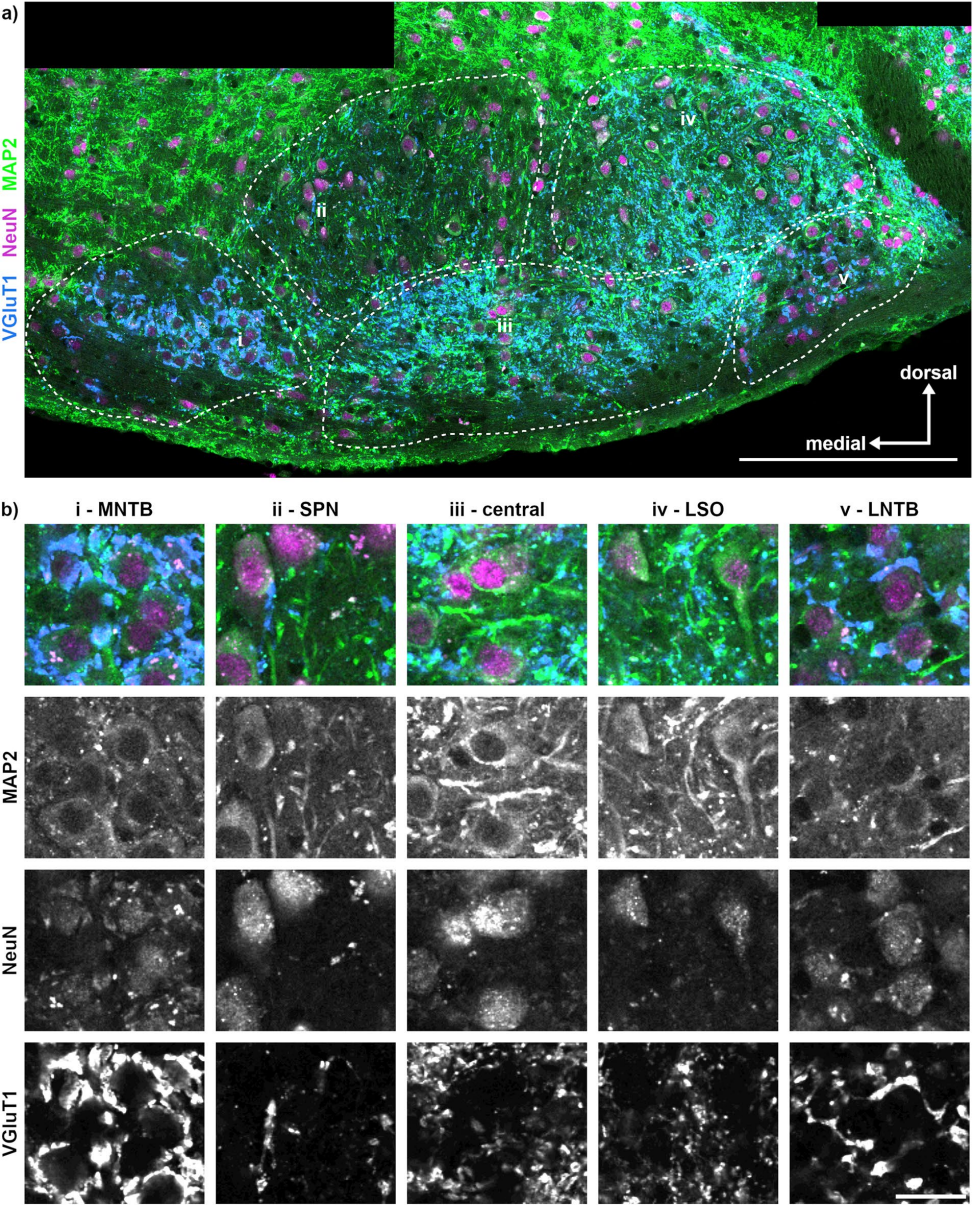
MAP2, NeuN and VGluT1 labelling in the SOC allows approximate identification of nuclei. (**a**) Overview of MAP2 (green), NeuN (magenta) and VGluT1 (blue) co-labelling in the SOC. The different SOC regions were delineated (dotted lines) and show from i (medial) to v (lateral): the medial nucleus of the trapezoid body (MNTB; i), superior paraolivary nucleus (SPN; ii), central SOC region (iii), lateral superior olive (LSO; iv) and the lateral nucleus of the trapezoid body (LNTB; v). The location of the labelling (i–v) corresponds to the region from which the images in b were taken. Scale bar equals 200 µm. (**b**) Magnifications of each identified region (i–v) shown as overlay (top row) with the corresponding gray scaled images depicted vertically. Scale bar equals 20 µm.

To seek identification and location of neuronal populations, we first used the neuronal post-synaptic markers MAP2 and NeuN in combination with VGluT1 that was used as a structural marker. With this combination, we identified in the SOC (from medial to lateral): MNTB, SPN, a central region, LSO and LNTB (Fig. 2a). The central region could not be further segregated and might be composed of a more heterogeneous cell population possibly including MSO and VNTB neurons. In the SOC, MAP2 immunofluorescence was weakest in somata and proximal dendrites of MNTB and LNTB neurons (Fig. 2a, b). In the SPN, the central region and the LSO, MAP2 immunofluorescence clearly labelled somata and proximal dendrites. NeuN immunofluorescence was present throughout the SOC neurons with variable intensity labelling their nucleus and soma (Fig. 2a, b).

All annotated SOC regions receive glutamatergic synaptic input detected by VGluT1 labelling (Fig. 2b). In the MNTB, large somatic VGluT1-positive synapses, presumably calyces of Held, were present. Sparse VGluT1 labelling dorsolateral to the MNTB is in accord with earlier identifications of the SPN^22,41^. Within the central region, small VGluT1 labelled synaptic structures were observed, but did not support further subdivision of this region (Fig. 2a, b). The LSO displays moderate VGluT1 labelling. Large somatic VGluT1-positive synapses identified the LNTB on the ventrolateral edge of the SOC (Fig. 2b).

**Figure 3:**
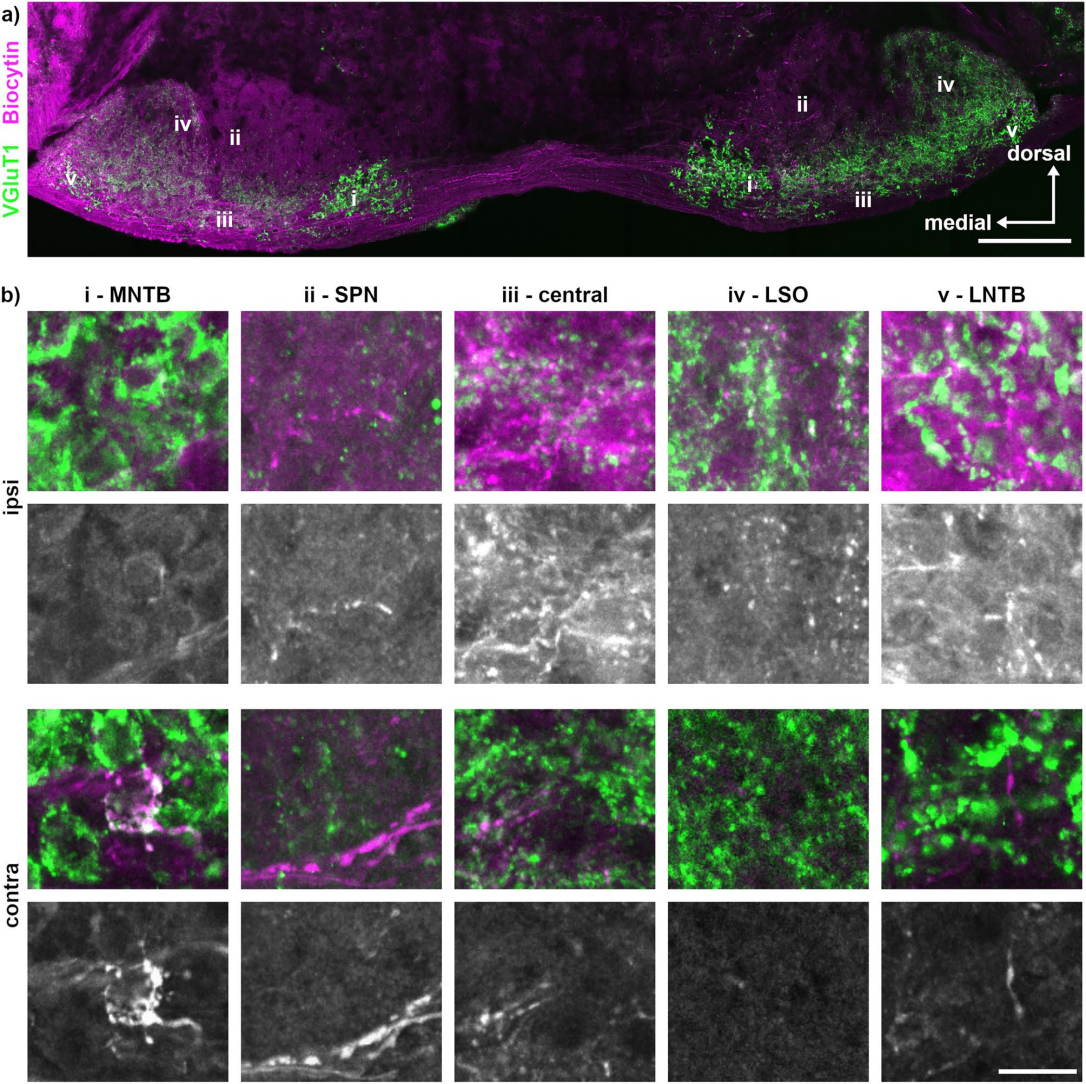
Biocytin tracing in Etruscan shrew. (**a**) Overview of ventral brainstem after biocytin injection (magenta) in the left cochlear nucleus (ipsilateral) and VGluT1 co-labelling (green). Previously identified nuclei are marked alongside by i–v. The location of the labelling (i–v) corresponds to the region from which the images in b were taken. Scale bar equals 200 µm. (**b**) Magnifications of the different ipsi- and contralateral SOC regions (i–v) shown as overlay. Below each overlay are the gray scale images of the tracer. Scale bar equals 20 µm.

To verify this initial identification, we seek insights into the input patterns originating from the cochlear nucleus, by biocytin labelling (Fig. 3). Ipsilateral applied biocytin labelled a large fiber bundle on the ventral edge of the section that crossed the midline (Fig. 3a). Ipsilaterally, biocytin labelling was observed in the SPN, the central region, the LSO and the LNTB but hardly in the MNTB (Fig. 3b). Contralaterally, in the MNTB, calyx terminals were visible. Furthermore, contralateral inputs were observed in the SPN, the central region and the LNTB. These input patterns corroborated our previous SOC annotations, as they are in line with the known mammalian circuitry^42^.

**Figure 4:**
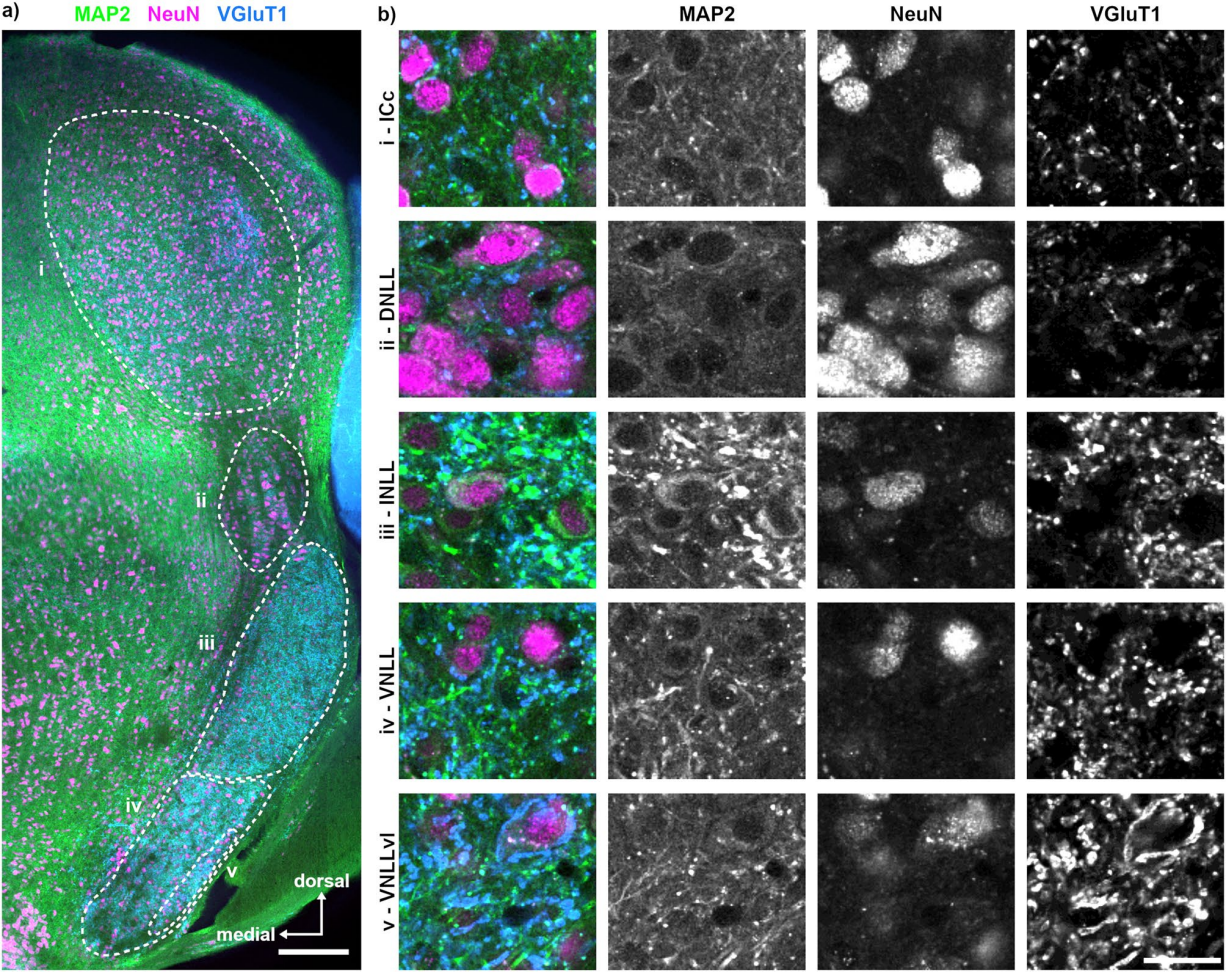
MAP2, NeuN and VGluT1 labelling in the NLL and IC supports the nuclei identification. (**a**) Overview of MAP2 (green), NeuN (magenta) and VGluT1 (blue) co-labelling in the LL and IC. Nuclei were delineated (dotted lines), showing from dorsal (top; i) to ventral (bottom; v): central nucleus of the inferior colliculus (ICc; i), the dorsal nucleus of the LL (DNLL; ii), the intermediate nucleus of the LL (INLL; iii), the ventral nucleus of the LL (VNLL; iv) and the ventrolateral part of the VNLL (VNLLvl; v). The location of the labelling (i–v) corresponds to the region from which the images in b were taken. Scale bar equals 200 µm. (**b**) Magnifications of each identified region (i–v) shown as overlay (left column) with the corresponding gray scaled images depicted horizontally. Scale bar equals 20 µm.

Since the co-labelling of structural markers with VGluT1 successfully identified SOC nuclei, we continued to determine the presence of known nuclei in the LL and the IC by this labelling approach (Fig. 4). The ICc could be distinguished by the intense and dense NeuN and VGluT1 labelling, whereas MAP2 labelling appeared less intense (Fig. 4b). Ventral to the IC, minor VGluT1, strong NeuN and faint MAP2 labelling indicated the position of the DNLL (Fig. 4b) matching the Nissl staining. Compared to the DNLL, the INLL and the VNLL have stronger VGluT1 labelling. In these structures, NeuN labelled cell nuclei and somata, while MAP2 labelling was more distinct and localized at somata and dendrites (Fig. 4b). More intensive NeuN labelling occurred in the VNLL compared to the INLL (Fig. 4a, b). The VNLLvl was clearly distinguishable by its big, somatic glutamatergic endbulb synapses (Fig. 4b), specific for this cell population^20,21^.

VGluT1 labelling supported the identification of the different auditory nuclei in Etruscan shrew, consistent with the expression pattern in other mammals. Therefore, we employed VGluT1 labelling as reference marker for further co-labelling.

**Figure 5:**
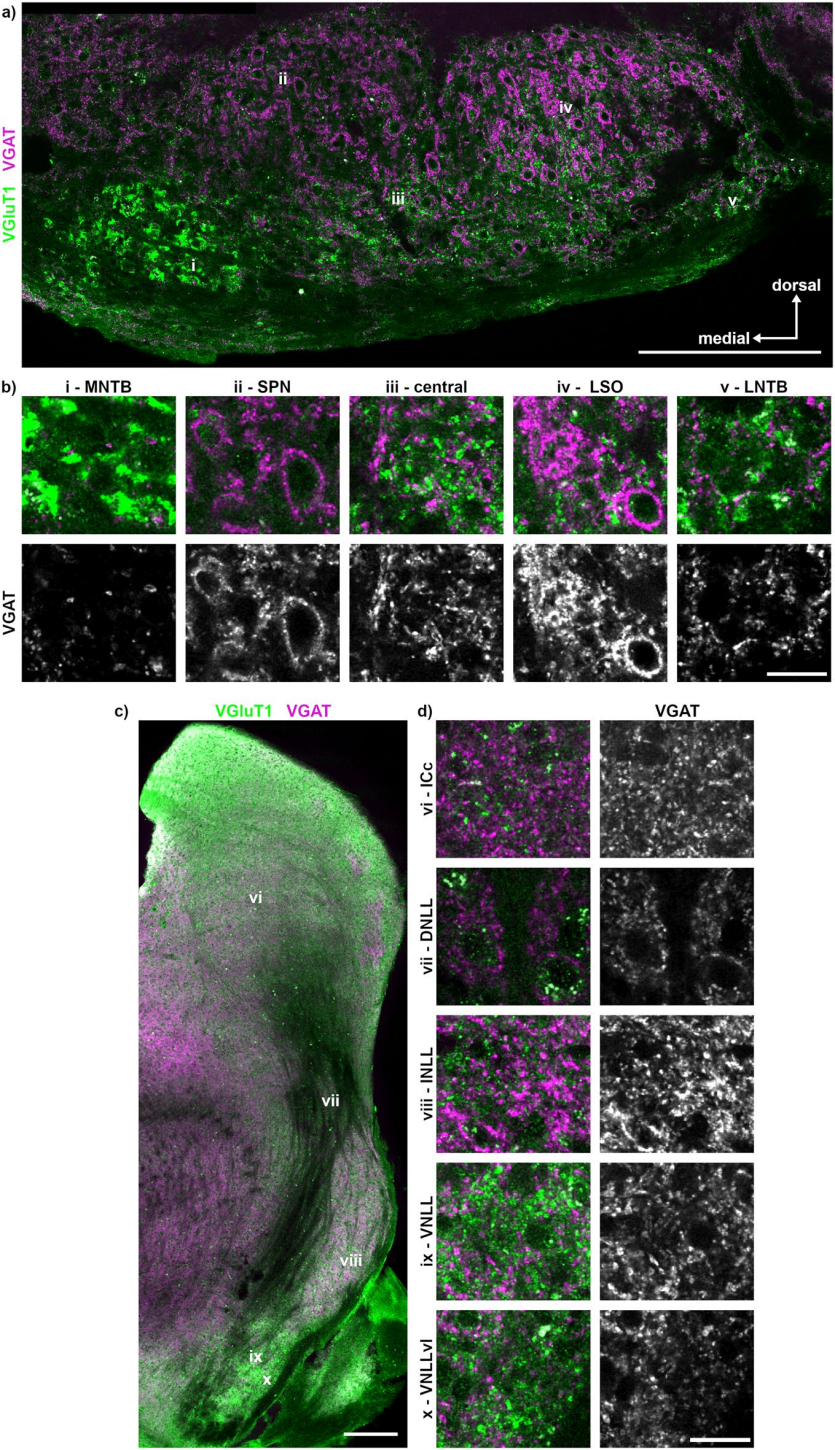
Distribution of inhibitory synapses within the SOC, NLL and IC. (**a**) Overview of the SOC showing the previously identified nuclei (i–v) with VGAT (magenta) and VGluT1 (green) labelling. The location of the labelling (i–v) corresponds to the region from which the images in b were taken. Scale bar equals 200 µm. (**b**) Magnifications of the different SOC regions (i–v) shown as overlay (top row) with the corresponding gray scaled VGAT images. Scale bar equals 20 µm. (**c**) Overview of the previously identified nuclei (vi–x) of the NLL and the IC. The location of the labelling (vi–x) corresponds to the region from which the images in d were taken. Scale bar equals 200 µm. (**d**) Magnifications of the ICc and the NLL (vi–x) shown as overlay (left column) with the corresponding gray scaled VGAT images. Scale bar equals 20 µm.

Next to the distribution of excitatory inputs, the distribution of inhibitory synapses was assayed by VGAT labelling (Fig. 5). We used this antibody directed to detect inhibitory synapses in general, because trials with various GlyT2 antibodies were unsuccessful to label specific glycinergic synapse. In the MNTB, the intensity and density of the VGAT labelling was lowest compared to other SOC nuclei (Fig. 5a, b). In the SPN and LSO, VGAT labelling surrounded somata, while in the central region and the LNTB it appeared more disbursed. Throughout the NLL and the IC, VGAT labelling was present and appeared of high intensity in the INLL (Fig. 5c, d).

### Distribution of inhibitory neurotransmitters

**Figure 6:**
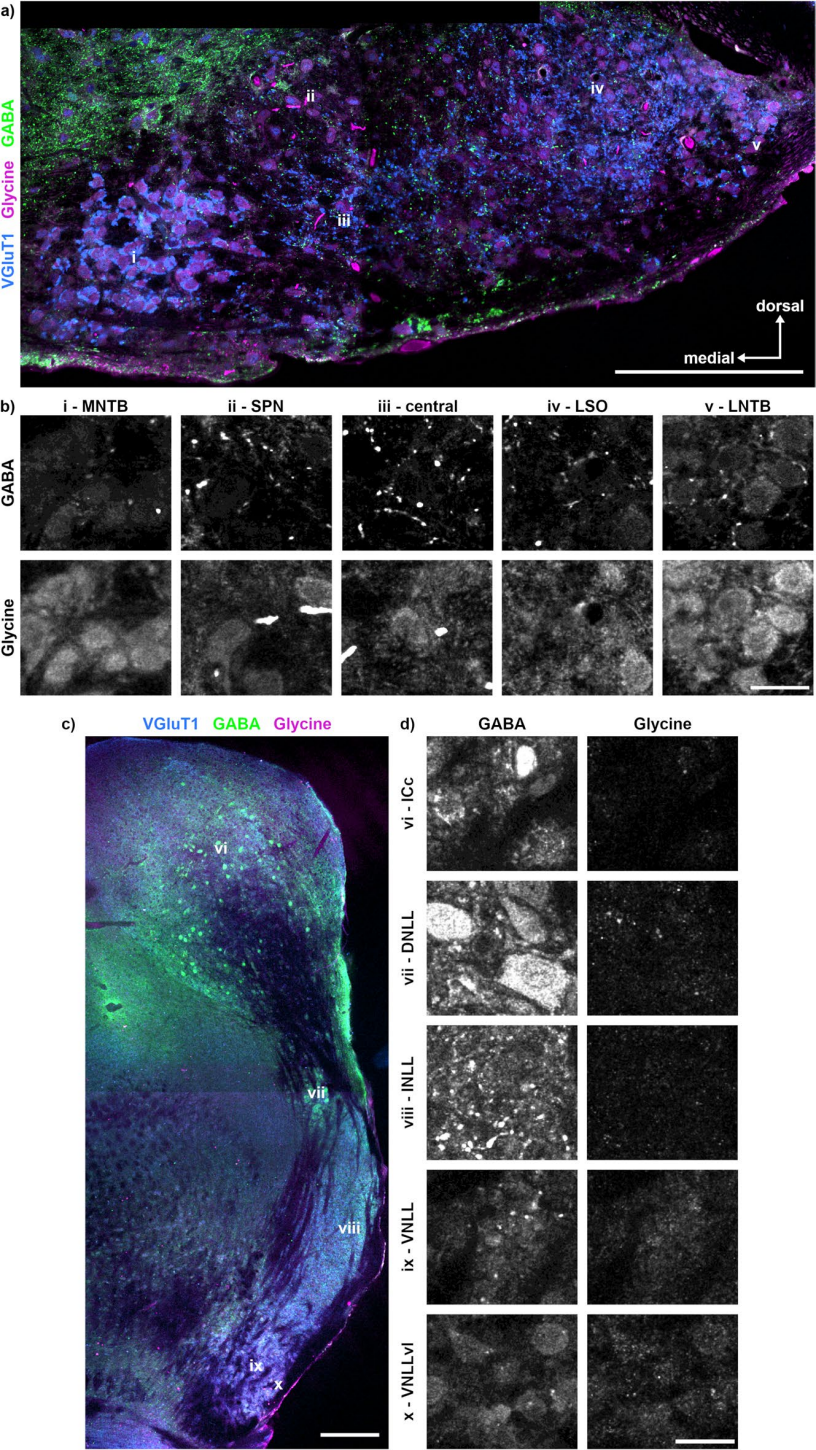
Distribution of GABA and glycine within the SOC, NLL and IC. (**a**) Overview of the previously identified nuclei (i–v) of the SOC with GABA (green), glycine (magenta) and VGluT1 (blue) labelling. The location of the labelling (i–v) corresponds to the region from which the images in b were taken. Scale bar equals 200 µm. (**b**) Magnifications of the different SOC regions (i–v) for GABA and glycine. Scale bar equals 20 µm. (**c**) Overview of the previously identified nuclei (vi–x) of the NLL and the IC. The location of the labelling (vi–x) corresponds to the region from which the images in d were taken. Scale bar equals 200 µm. (**d**) Magnifications of the ICc and the NLL (vi – x) for GABA and glycine. Scale bar equals 20 µm.

Next to the distribution of synaptic input types, the transmitter content of neurons is an indicator of nucleus identity. Therefore, we labelled sections with antibodies targeted against GABA and glycine (Fig. 6). Glycinergic neurons were found in all SOC nuclei, yet dominating in the MNTB, SPN and LNTB. In the MNTB and LNTB, a partial GABA co-labelling was observed (Fig. 6b). GABA labelling yielded punctate structures in the SPN, central region, LSO and LNTB that might indicate GABAergic synaptic inputs to these neurons. In the NLL and IC, inhibitory neurons were mainly GABAergic with some glycinergic co-labelling in the VNLLvl (Fig. 6c, d). DNLL neurons were clearly GABA-positive (Fig. 6d). In the INLL, no GABAergic somata but punctate structures reminiscence of buttons were observed (Fig. 6d). Overall, we observed a ventrodorsal increase and decrease of GABAergic and glycinergic labelling, respectively, in the NLL. Moreover, the lack of somatic glycine and GABA labelling in various structures indicates antibody specificity.

### Distribution of CaBPs

**Figure 7:**
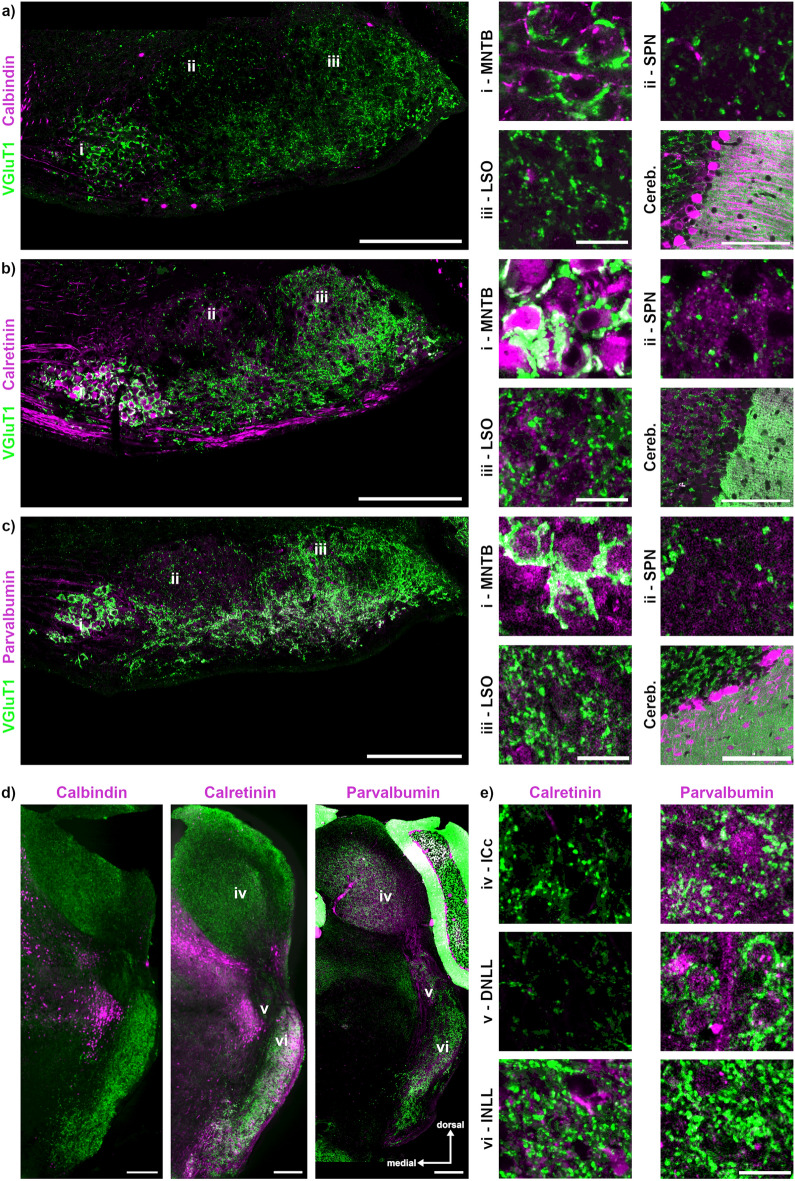
Distribution of calcium binding proteins within the SOC, NLL and IC. (**a**) Left: Overview of SOC labelled with calbindin (CB; magenta) and VGluT1 (green), i–iii mark the nuclei shown as insets on the right. The location of the labelling corresponds to the region from which the images to the right were taken. Right: Magnifications from MNTB (i), SPN (ii), LSO (iii) and cerebellum (Cereb.). Cerebellum image was taken from the same section. Scale bars equal 200 µm (overview), 20 µm (SOC magnifications) and 100 µm (Cereb.). (**b**) Same as in (a) for calretinin (CR). (**c**) Same as in (a) for parvalbumin (PV). (**d**) Overview of sections containing the NLL and IC labelled with CB, CR or PV (magenta) and VGluT1 (green), numbers (iv–vi) alongside the magnified nuclei in e. The location of the labelling corresponds to the region from which the images in e were taken. Scale bars equal 200 µm. (**e**) Magnifications of the ICc (iv), DNLL (v) and INLL (vi) for CR and PV (magenta) with VGluT1 (green) co-labelling. Scale bar equals 20 µm.

CaBPs influence neuronal signalling^43–46^. In the auditory brainstem, their differential presence was used to identify nuclei localisation and development^47–52^ as well as projection patterns^53^. The expression patterns of CB, CR and PV are species-dependent^22,48,49,51,52^. Here, we co-labelled CB, CR and PV together with VGluT1 (Fig. 7).

Somatic CB labelling was nearly absent in the SOC (Fig. 7a). Exemplified for the MNTB, SPN and LSO, some labelling of CB occurred in the neuropil (Fig. 7a). However, this supposedly synaptic labelling did not co-localize with VGluT1, indicating a possible inhibitory calbindinergic input. To illustrate that the absence of CB from the SOC is not dependent on the antibody, we used cerebellar labelling as a control. As expected from other species^22,54^, Purkinje neurons were CB-positive (Fig. 7a). In the SOC, CR-positive somata were restricted to the MNTB and LNTB (Fig. 7b). In some cases, CR labelling co-localized with VGluT1-positive synapses in the MNTB. In the SPN and LSO, CR labelled non-VGluT1-positive, punctate structures in the neuropil (Fig. 7b), suggesting calretinergic inhibition, possibly originating from CR-positive neurons of the MNTB. CR-positive labelling of the neuropil was also detected in the cerebellum (Fig. 7b). PV labelling was weak but distributed similarly to CR labelling in the SOC (Fig. 7c). Some weak pre- and postsynaptic PV labelling was detected in the MNTB and non-VGluT1 co-localizing puncta in the SPN and LSO. To corroborate this finding, our approach yielded strong PV-positive signals in cerebellar Purkinje cells, as has been described for other species^54,55^.

In the NLL and the IC, CB was not detected by immunofluorescence, yet, it is present in the medial paralemniscal zone (Fig. 7d). Somatic CR labelling was nearly absent in the IC and DNLL, but found in the lateral parts of the INLL and VNLL and the medial paralemniscal zone (Fig. 7d). Sparse neuropil labelling of CR occurred in the ICc and INLL (Fig. 7e). PV labelling appeared present in fibers passing the NLL and the IC (Fig. 7d). Some somata were labelled in the ICc and very weakly in the DNLL (Fig. 7e). Taken together, weak somatic CaBP expression was restricted to the MNTB, LNTB, lateral INLL, DNLL and IC, while it was absent in SPN, central region, LSO, VNLLvl and large parts of VNLL and INLL.

### Distribution of HCN1 and Kv1.1 channels

**Figure 8:**
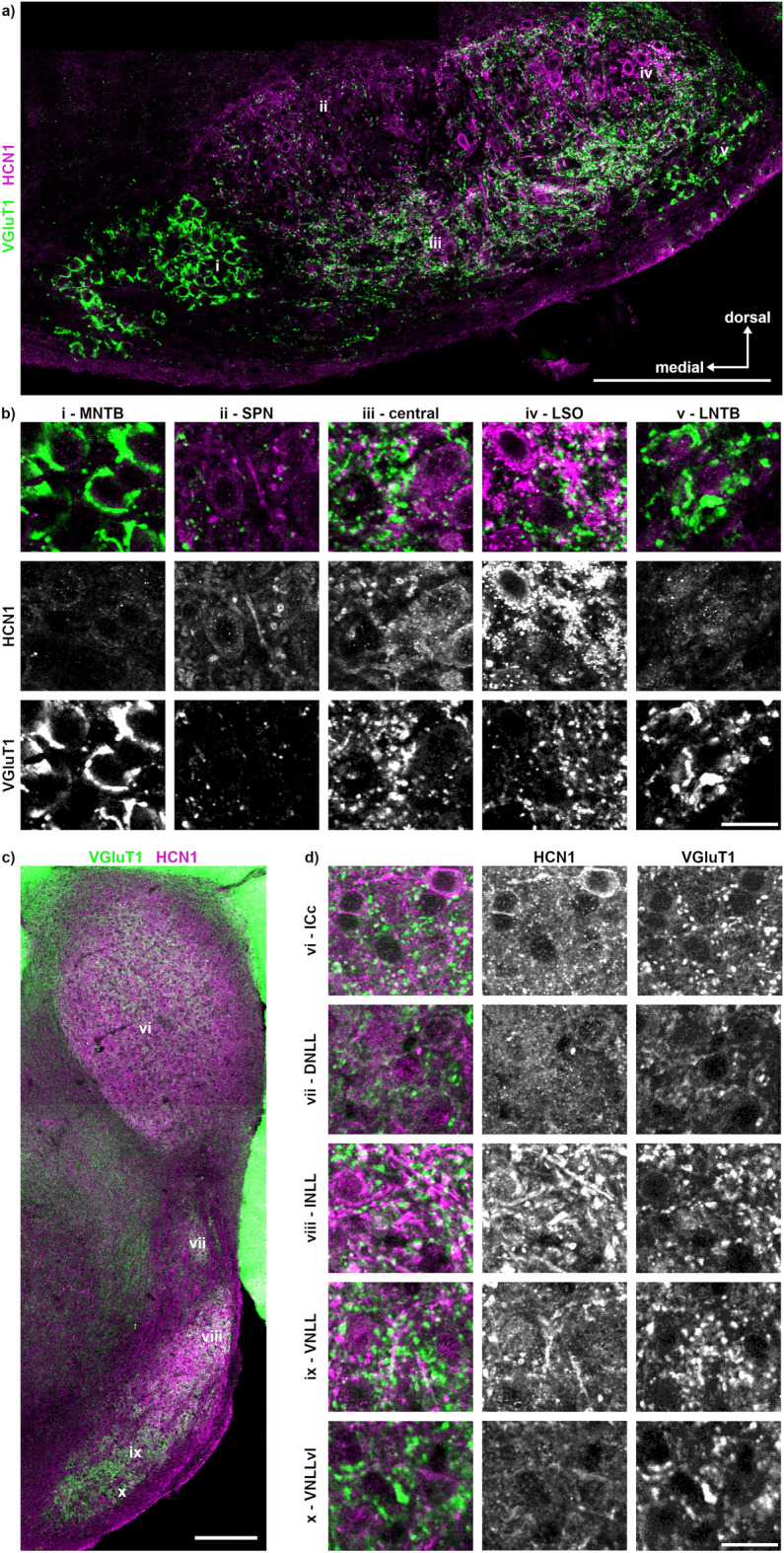
Distribution of HCN1 within the SOC, NLL and IC. (**a**) Overview of the previously identified nuclei (i–v) of the SOC with HCN1 (magenta) and VGluT1 (green) labelling. The location of the labelling (i–v) corresponds to the region from which the images in b were taken. Scale bar equals 200 µm. (**b**) Magnifications of the different SOC regions (i–v) shown as overlay (top row) with the corresponding gray scaled images below. Scale bar equals 20 µm. (**c**) Overview of the previously identified nuclei (vi–x) of the NLL and the IC. The location of the labelling (vi–x) corresponds to the region from which the images in d were taken. Scale bar equals 200 µm. (**d**) Magnifications of the ICc and the NLL (vi–x) shown as overlay (left column) with the corresponding gray scaled images to the right. Scale bar equals 20 µm.

In the Etruscan shrew’s SOC, HCN1-positive labelling was present in the SPN, the central region and especially in the LSO and nearly absent in the MNTB and LNTB (Fig. 8a, b). HCN1 did not co-label with VGluT1 and, together with the labelled structures, we interpret HCN1 location as mainly somatic and dendritic. In the NLL, HCN1-positive labelling was observed in the VNLL, INLL and ICc but not in the VNLLvl and DNLL (Fig. 8c, d). In the ICc, some neurons were positively labelled for HCN1, indicating a heterogeneous expression pattern.

**Figure 9:**
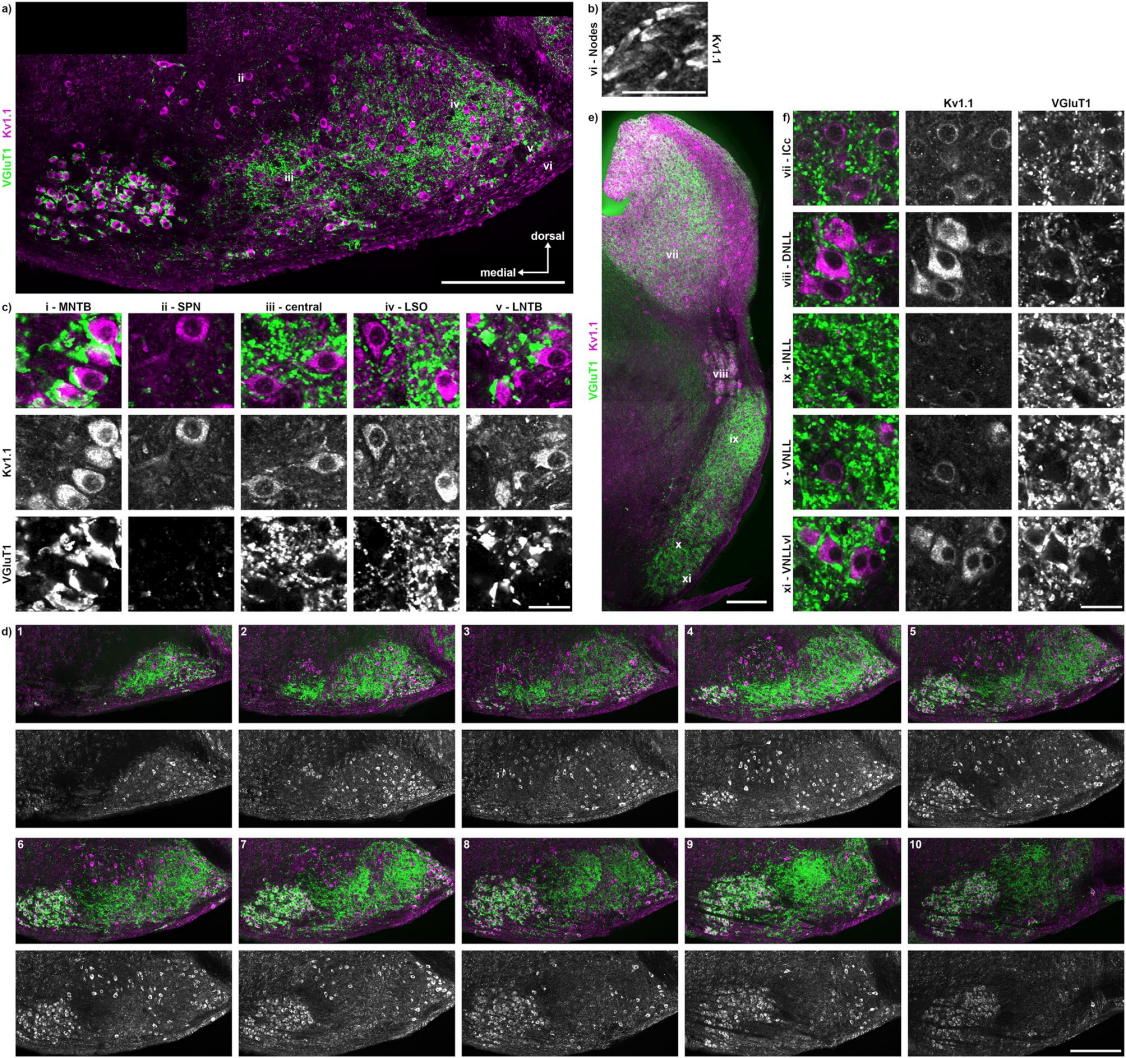
Distribution of Kv1.1 within the SOC, NLL and IC. (**a**) Overview of the previously identified nuclei (i–v) of the SOC with Kv1.1 (magenta) and VGluT1 (green) labelling. The location of the labelling (i–v) corresponds to the region from which the images in c were taken. Approximate location of the magnification shown in b is marked with the number vi. Scale bar equals 200 µm. (**b**) Magnification of nodes of Ranvier (vi) within the fiber bundle ventral to the LNTB (v). Scale bar equals 20 µm. (**c**) Magnifications of the different SOC regions (i–v) shown as overlay (top row) with the corresponding gray scaled images below. Scale bar equals 20 µm. (**d**) Serial sections of Kv1.1 and VGluT1 labelled SOC region. Number of the sections is given in the top left corner of the overlay images, every 50 µm is shown. Kv1.1 labelling is also shown below in gray scale. Scale bar equals 200 µm. (**e**) Overview of the previously identified nuclei (vii – xi) of the NLL and the IC. The location of the labelling (vii–xi) corresponds to the region from which the images in f were taken. Scale bar equals 200 µm. (**f**) Magnifications of the ICc and the NLL (vii–xi) shown as overlay (left column) with the corresponding gray scaled images to the right. Scale bar equals 20 µm.

In Etruscan shrew, Kv1.1 immunofluorescence was detected throughout the SOC neurons (Fig. 9a) and in the juxta-nodal region of nodes of Ranvier (Fig. 9b). Within each SOC nucleus, Kv1.1 labelling was strong in the somata and weaker in the dendrites and did not overlay with glutamatergic synapses (Fig. 9c). Since the Kv1.1 labelling reliable detects neurons and VGluT1 supports nuclei identification in the SOC, we used this antibody combination in serial sections (*n* = 2) to highlight the major nuclei and their caudorostral appearance (Fig. 9d). The LSO and LNTB started most caudally and was followed by the SPN, and finally by the MNTB. The MNTB extended most rostrally compared to the other nuclei. In the serial section, the central region did not highlight specific cell populations with distinct organization (Fig. 9d). All neurons in the DNLL appeared intensely labelled (Fig. 9e). In the INLL and VNLL, Kv1.1 labelling was either absent or weaker compared to other LL structures (Fig. 9f). In the VNLLvl and in a subpopulation of ICc neurons, Kv1.1 labelling was detected. Our labelling indicates a predominant post-synaptic localisation of Kv1.1, because of its shape and the nearly absence of co-labelling with VGluT1.

To give an estimate about the size of auditory nuclei in the Etruscan shrew, we measured the cross-section area of the MNTB, LSO and DNLL. Extracted from different labellings the size of the MNTB was 0.034 ± 0.001 mm^2^ (*n* = 45), from the LSO 0.05 ± 0.001 mm^2^ (*n* = 46) and from the DNLL 0.05 ± 0.018 mm^2^ (*n* = 34). This compares to about a 3 and fivefold smaller size to the MNTB and LSO of gerbil respectively^56^.

## Discussion

**Figure 10:**
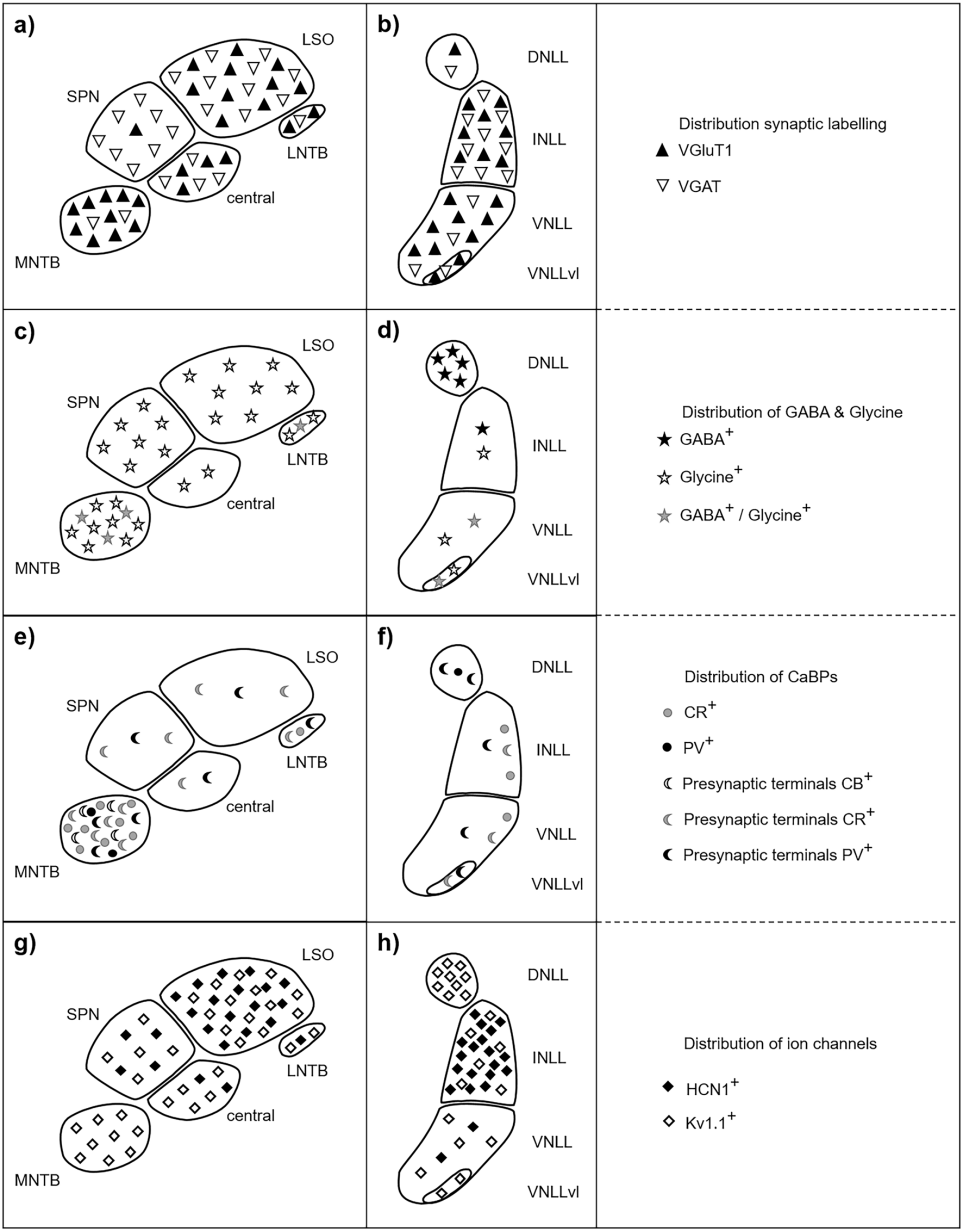
Schematic summary of the probed expression patterns in the SOC and NLL of the Etruscan shrew. Explanations of the symbols are given on the right. More symbols indicate an apparent higher intensity of labelling and does not necessarily reflect the percentage of labelled somata. (**a**, **b**) Distribution of synaptic labelling. (**c**, **d**) Distribution of GABA- and glycinergic cells. (**e**, **f**) Distribution of CaBPs. (**g**, **h**) Distribution of HCN1 and Kv1.1 channels.

Here, we describe the architecture of the SOC and the LL and therein the expression patterns of marker proteins in the Etruscan shrew summarized in Fig. 10. The combination of immunofluorescently detected protein expression and Nissl staining allows us to identify in the SOC the MNTB, SPN, LSO and LNTB while a MSO and a VNTB could not unambiguously be determined. In the LL, the three major nuclei and their subdivision (DNLL, INLL, VNLL, VNLLvl) were identified. The inhibitory and excitatory presence and distribution of synaptic terminals agrees with that of other mammals (Fig. 10a + b). A peculiarity exists in the apparent low expression level of CaBPs (Fig. 10e + f). All nuclei except the SPN share the known presence of transmitter types (Fig. 10c + d). HCN1 and Kv1.1 expression largely compared to rodents (Fig. 10g + h).

### Identification and arrangement of auditory nuclei

In the Etruscan shrew, the MNTB and LNTB appear as the major landmarks for the SOC by their densely packed globular cells readily apparent in Nissl staining. The neuronal markers NeuN and MAP2 helped little in further separating the SOC nuclei. Using the pre-synaptic markers VGluT1 and VGAT, the MNTB, SPN, LSO and LNTB could be distinguished. MNTB and LNTB were distinguished by the large somatic VGluT1-positive synapses, e.g.^41^, SPN by its predominant inhibitory input^22^ and the LSO by the mixture of VGluT1 and VGAT^22,57^. Yet again, no subdividing of the central region was feasible. The same difficulty for the central region was given by other markers such as voltage-gated ion channels, CaBPs or transmitters despite an overall heterogeneous appearance. Therefore, we conclude that the SOC in Etruscan shrew is dominated by the MNTB, SPN, LSO and LNTB and that other structures such as the MSO, VNTB and lateral and medial olivocochlear bundle cells are either strongly reduced, merged into a heterogeneous cell population or moved to unexpected positions. Since we were unable to untangle the cell population in the central SOC further, we termed it the central region.

In the LL, a well-stained Nissl region of globular neurons on the ventrolateral side stood out. This sub-region contained large VGluT1 labelled terminals on GABA and glycine co-labelled somata. Therefore, this region resembled the fast relaying cells that receive endbulb synapses^20,21^. Thus, similar to mice and some bats (*Phyllostomus discolor* and *Carollia perspicillata*), this cell population is located ventrolaterally in Etruscan shrew and differs in its location from bats like *Eptesicus fuscus*, *Rhinolophus ferrumequinum* or *Petronotus parnellii* where it is located either more medially, dorsally or ventrally^8^. Etruscan shrews are separated from rodents by 94 MYA and from bats by about 86 MYA^58^. Thus, the ancestral location of this cell population might be the ventrolateral position and further rearrangements in some bats might have followed other so far unknown adaptations.

From all three NLL, the DNLL can most easily be distinguished. The DNLL is marked by high intensity GABA and Kv1.1, low intensity VGluT1 labelling and the presence of pre- and partially post-synaptic PV labelling and matches therefore with other mammalians^22,59–62^. The border between INLL and VNLL is difficult to morphologically delineate^19,63^. However, the specific transmitter types of these nuclei seem to be the key feature for their separation^59,61,64^. In Etruscan shrew, we found that next to a difference in transmitter types a more intense VGAT, CaBP and HCN1 labelling in the INLL compared to the VNLL additionally helped to separate these structures.

In conclusion, the Etruscan shrew’s auditory brainstem is overall similar to other mammals, yet with specific differences. First, the MSO could not be unambiguously identified. This is in line with the concept that the small heads of terrestrial mammals are not suited to detect interaural time differences and that this processing task is the key feature of the MSO. Second, the INLL and VNLL could well be separated by a distinct pattern of markers. Each of the several markers indicated slight differences that accumulated into a clear distinction that is supported by the known difference in neurotransmitter content. Third, the arrangement of the VNLLvl follows that of rodents and some bats and therefore likely reflects the ancestral one.

### Cellular distribution of inhibitory neurotransmitter

The distribution of cells with GABA- and glycinergic neurotransmitter content appear to largely match that of other mammals^59–61,64–68^. One difference was the glycinergic and not GABAergic transmitter content of SPN neurons. The SPN is regarded to be GABAergic or mixed GABA/glycinergic for example in rat, guinea pig, gerbil or bat^65–68^. The glycinergic cell population of the SPN in Etruscan shrew agrees more with macaques, where a similar area of the SOC lacks GAD but contains glycinergic neurons^60^. Thus, despite being generally inhibitory, this cell population might use different transmitters, irrespectively of head size.

### Functional considerations

Labelling of transmitter specific presynaptic structures allows the detection of input patterns of nuclei. VGluT1 and VGAT immunofluorescence thereby discriminates between excitatory and inhibitory inputs. In Etruscan shrew, the gross input patterns in auditory brainstem nuclei are similar to that of other mammals^22,41,57,62,69^. Thus, we consider the overall circuit architecture of the auditory system conserved in Etruscan shrews.

In the auditory brainstem, the presence of Kv1.1 and HCN1 channels are functionally important. In Etruscan shrew, Kv1.1 channel distribution matches that of other mammals^22,33^. Interestingly, the expression pattern of Kv1.1 is similar in DNLL and MNTB neurons. In MNTB neurons, Kv1.1 is implicated in supporting onset action potential firing by suppression excitability^33^. DNLL neurons in rodents have been shown to fire continuously to current injections^70,71^. Whether in the DNLL ongoing firing behavior is absent in Etruscan shrews or these neurons use Kv1.1 for different purposes is so far not explored and cannot be answered by histology. In the auditory brainstem of Etruscan shrew, HCN1 channels are similarly distributed to gerbils, naked mole rat and mice^57,72^ but differ from bats where MNTB neurons are HCN1-positive^22^. The heterogeneous expression of HCN1 in the INLL of Etruscan shrew would suggest that some neurons either generate short integration times or off-set inhibition. Whether this heterogeneity matches the reported heterogeneity of cell morphology^19,62,63^ is unresolved so far.

Calcium binding proteins are endogenous calcium buffers. The largely soluble CaBPs CB, CR and PV are implicated in shaping calcium signals relevant for synaptic transmission^44,45^ and dendritic calcium signaling^43,46^. In fast spiking neurons, CaBPs can be considered to buffer the excess calcium influx generated by action potential sequences. In the auditory brainstem of many mammals, these proteins are highly expressed in agreement with the high firing rates. Thereby, the CaBP expression patterns differ between mammals^22,48,49,73^. In Etruscan shrew, the low expression or absence of CaBPs is peculiar. This limited presence of CaBPs is expected to influence calcium signaling. For example, synaptic short-term plasticity in auditory circuits might be biased to facilitation in Etruscan shrew rather than depression as in other mammals^74^. For viability, in Etruscan shrew to restrict intracellular free calcium accumulation either other CaBPs are expressed as buffers, calcium channel densities are lower or extrusion rates are faster compared to other mammals.

Our labelling of different marker and functional proteins enables the differentiation of the main auditory brainstem nuclei in the SOC and LL in the smallest terrestrial mammal and gives insights into their functionality. For the SOC, the main nuclei are the MNTB, SPN, LSO and LNTB. For the LL, the VNLL, VNLLvl, INLL, DNLL and the paralemniscal region are highlighted. We argue that the head size and the peripheral auditory structures are in agreement with ancestral mammalian features. These constrains will hinder sound source location by interaural time differences and thereby allow for a reduction of the MSO in Etruscan shrews. The presence of the MSO in exclusively high frequency listening mammals such as bats can be explained by their suggested differences in circuitry and functionality^75^. Thus, the Etruscan shrew might serve as system to illuminate the ancestral mammalian auditory brainstem structures relevant for exquisite hunters.

## Methods

### Experimental animals

In this study, we included 37 between three and 22 months old Etruscan shrews (*Suncus etruscus*) of both sexes, bred in the institute’s colony. The shrews were housed in terraria containing a layer of dry soil, moss, stones, pieces of wood and small flowerpots. Their diet consisted of field crickets (every second day) and water ad libitum. Light cycle was constant throughout the year with 12 h of light and dark phase each. Ambient temperature was maintained at ~ 26 °C and relative humidity at 26 ± 0.6% (mean ± SEM).

### Brain Tissue Preparation

All experiments were in accord with ARRIVE guidelines, the national laws and local regulations and were approved by the animal welfare officer on the local animal protocol TiHo-T-2021–4. Animals were euthanized with CO_2_ and declared dead after ~ 2 min of breathing arrest. Animals were perfused with Ringer-Heparin solution followed by either 4% paraformaldehyde (PFA) in phosphate buffered saline (PBS) for Nissl staining or different PFA solutions for immunofluorescence labelling. Dependent on the primary antibody, we used 4% PFA, 3.7% PFA with 0.3% glutaraldehyde or 2% PFA with 15% picric acid in PBS (Table 1). The 2% PFA solution was used as standard, because it conserves more epitopes. Following perfusion, the brains were removed and post-fixed in the matching PFA solution over night at 4 °C.

**Table 1:** Primary antibodies.

Target	Host	Type	Dilution	PFA (%)	Used 2nd AB	Company	RRID
Calbindin D-28 k	Mouse	Monoclonal	1:5000	2	Cy3	Swant (Cat#300)	AB_10000347
Calretinin	Mouse	Monoclonal	1:5000	2	Cy3	Swant (Cat#6B3)	AB_10000320
GABA	Mouse	Monoclonal	1:5000	3.7	A488, Cy3	Swant (Cat#3A12)	AB_2721208
Glycine	Rabbit	Polyclonal	1:1500	3.7	Cy3	MoBiTec (Cat#1015GE)	AB_2560949
HCN1	Rabbit	Polyclonal	1:400	2	Cy3	Synaptic systems (Cat# 338 003)	AB_2620083
Kv1.1	Rabbit	Polyclonal	1:2500	2	A488, Cy3	Alomone labs (Cat# APC-009)	AB_2040144
MAP2	Chicken	Polyclonal	1:3000	2	AMCA, A488	Neuromics (Cat# CH22103)	AB_2314763
NeuN (D4G4O) XP	Rabbit	Monoclonal	1:400	2	Cy3	Cell Signaling technology (Cat# 24307)	AB_2651140
Parvalbumin	Mouse	Monoclonal	1:5000	4	A488, Cy3	Swant (Cat# 235)	AB_10000343
VGAT	Guinea pig	Monoclonal	1:8000	2	A488, Cy3	Synaptic systems (Cat# 131 308)	AB_2832243
VGluT1	Guinea pig	Polyclonal	1:2000	2, 3.7, 4	A488, A647, Cy3	Synaptic systems (Cat# 135 304)	AB_887878
VGluT1	Mouse	Monoclonal	1:1000	2	A488, A647	Synaptic systems (Cat# 135 311)	AB_887880

For biocytin labelling, animals were deeply anesthetised with isoflurane before decapitation. After brain removal, a small biocytin crystal (B4261, Sigma-Aldrich) was pushed onto the cochlear nucleus. The brains containing biocytin were incubated for 3 h in artificial cerebrospinal fluid (125 mM NaCl, 25 mM NaHCO_3_, 2.5 mM KCl, 1.25 mM NaH_2_PO_4_, 1 mM MgCl_2_, 1.2 mM CaCl_2_, 25 mM glucose, 0.4 mM ascorbic acid, 3 mM myo-inositol and 2 mM Na-pyruvate, pH 7.4), saturated with 95% O_2_ and 5% CO_2_ at 33–35 °C. Brains were fixed in 2% PFA with 15% picric acid in PBS over night at 4 °C. For immunofluorescence co-labelling, tissue was embedded in 4% agar in deionized water and 50 µm transversal sections were cut with a vibratome (7000smz-2, Campden) at room temperature.

For the Nissl staining, transversal sections were cut with a cryostat (HM 500 OM, Microm) after cryoprotection in 15% and 30% sucrose dissolved in PBS at 4 °C each at least for 24 h. As tissue freezing medium, a solution of 2% gelatine in deionized water was used. The slicing temperature was set to −20 °C and the section thickness to 30 µm.

### Nissl staining and immunofluorescence labelling

Nissl staining (*n* = 4) was carried out with a standard protocol with on slides mounted sections using incubation times of 22 min in the cresyl violet staining solution. For free-floating immunofluorescence (*n* = 33), an aldehyde-reduction protocol for background reduction was used. After three PBS washes, the sections were incubated on ice in NaBH_4_ (1 g/l) dissolved in PBS four times each 10 min as aldehyde reduction step. After washing three times in PBS, sections were blocked for 30 min in blocking solution containing 0.5% triton v/v, 1% bovine serum albumin w/v and 0.1% saponin w/v in PBS. Sections were incubated in blocking solution containing primary antibodies (Table 1) for 72 h at 4 °C. Before incubation with the respective secondary antibodies (s. Table 2), sections were washed in washing solution (0.5% triton v/v and 0.1% saponin w/v in PBS) three times for 10 min. The secondary antibodies were diluted in blocking solution and incubated for 4 h at room temperature. Afterwards, the sections were washed with washing solution (three times 10 min) and transferred to PBS, before mounting on gelatinized slides with Vectra shield and sealed with nail polish. To fix GABA and glycine more effective, a PFA solution with glutaraldehyde was used, since this binds stronger to amino groups. For the PV labelling, the PFA solution was switched to 4% according to the literature ^76^. The following amounts of sections from at least three animals with the age range given in Table 3 were used for each antibody combination: biocytin + VGluT1 *n* = 16/9 (SOC/NLL); MAP2 + NeuN + VGluT1 *n* = 32/16; VGAT + VGluT1 *n* = 32/16; GABA + glycine + VGluT1 *n* = 20/14; CB + VGluT1 *n* = 18/10; CR + VGluT1 *n* = 18/10; PV + VGluT1 *n* = 40/20; HCN1 + VGluT1 *n* = 14/6; Kv1.1 + VGluT1 *n* = 42/21.

**Table 2:** Secondary antibodies.

Antigen	Conjugate	Host	Dilution	Company	Catalogue number	RRID
Chicken	Alexa488	Donkey	1:300	Dianova	703-546-155	AB_2340376
Guinea pig	Alexa488	Donkey	1:800	Dianova	706-546-148	AB_2340473
Mouse	Alexa488	Donkey	1:200	Dianova	715-545-150	AB_2340846
Rabbit	Alexa488	Donkey	1:800	Dianova	711-545-152	AB_2313584
Guinea pig	Alexa647	Donkey	1:200	Dianova	706-606-148	AB_2340477
Mouse	Alexa647	Donkey	1:200	Dianova	715-606-150	AB_2340865
Chicken	AMCA	Donkey	1:100	Dianova	703-156-155	AB_2340362
Biotin	Cy3	*Streptomyces avidinii*	1:500	Dianova	016-160-084	AB_2337244
Guinea pig	Cy3	Donkey	1:800	Dianova	706-165-148	AB_2340460
Mouse	Cy3	Donkey	1:200	Dianova	715-165-151	AB_2315777
Rabbit	Cy3	Donkey	1:800	Dianova	711-165-152	AB_2307443

**Table 3:** Animal age used in the different labelling.

Labelling	# of animals	Age range [months]	Age average [months]
Nissl	4	3–22	10.25
MAP2 + NeuN	4	7–14	10.5
Biocytin	3	11–18	14.33
VGAT	3	8–21	14.33
GABA + Gly	3	3–6	4.33
CB	5	5–10	8.8
CR	5	5–10	8.8
PV	4	7–18	13.25
HCN1	3	6–19	11.67
Kv1.1	5	9–18	12.8
VGluT2	3	7–14	11.33

### Antibody characterisation

The reactivity of most primary antibodies has not be tested in the Etruscan shrew so far, apart from a description of CB^77,78^ and PV^76^. The following description of labelling feasibility and specificity shows that the used antibodies have been successfully applied in brain sections of other species.

Within the auditory brainstem, anti-CB labels somata and neuropil in geckos^79^, gerbils^53^ and bats^22^. The anti-CB antibody was purified from chicken gut^80^. It was tested for specificity in various mammals by western blot and by knock-out mice (company description). In the Etruscan shrew, this antibody was successfully used in the hippocampus and cortex^77,78^.

The anti-CR antibody was produced against recombinant human calretinin-22 k in mice. It was tested for specificity in various animals via Western Blot and knock-out mice (company description). This anti-CR antibody labels neuronal structures in cat^81^, human^82^ and bat^22^.

The anti-GABA antibody was purified in mice and verified by ELISA^83^. In the auditory brainstem, this GABA antibody labels somata and neuropil in frogs^84^, chicken^85,86^ and gerbil^62^.

The anti-glycine antibody was raised in rabbit with conjugates of glycine-glutaraldehyde-carriers and verified by ELISA^87^. It has been successfully used in fish^88^ and labelled specifically somata of glycinergic neurons in the auditory brainstem of guinea pig^89^ and gerbil^62^.

The anti-HCN1 antibody was produced in rabbit against rat recombinant HCN1 (company description). Its reactivity was tested in rats and mice (company description) and labelled neuronal somata in bat^22^.

The anti-Kv1.1 antibody was raised against mouse KCNA1 (company description). It was verified by a knock-out mouse^90^ and by western blots (company description). In the auditory brainstem of bats, Kv1.1 labels somata, neuropil, and highlights nodes of Ranvier^22,35^.

The anti-MAP2 antibody was produced in chicken against the human recombinant protein and its reactivity was tested against humans, primates, mice and rats (company description). It labels neuronal somata and dendrites in neurons e.g., of gerbil^53,62^ and bat^35^.

The anti-NeuN antibody was raised against recombinant NeuN in rabbit and tested for reactivity in humans, mice and rats (company description). It is specifically expressed in the soma and cell nucleus of most neuronal cells of the brain^91^.

The anti-PV antibody was produced in mouse, tested for various species and applications (company description) as well as verified by knock-out^92^. It labels specifically neuronal structures in gecko^79^, rat^93^, bats^22^, hedgehogs^94^ and Etruscan shrews^76^.

The anti-VGAT antibody was purified against rat recombinant VGAT and was verified by knock-out (company description). It labels specifically inhibitory – namely GABAergic and glycinergic–synapses in at least mouse, rat and gerbil^62,95,96^.

The anti-VGluT1 antibody was produced in guinea pig against recombinant VGluT1 of rat, near the carboxy terminus (company description). It is verified by knock-out^97^ to label glutamatergic synapses in bat^22^, guinea pigs^98^ and gerbil^99^.

A second anti-VGluT1 antibody was raised in mice against the same locus than the latter and was also verified by knock-out (company description). It labels the glutamatergic synapses in the auditory brainstem at least in guinea pig and gerbil^62,100^.

### Image Acquisition and data analysis

The Nissl sections were photographed on a Light Therapy Lamp (model TT-CL011, Taotronics) using a Canon EOS 60D camera with an EFS 18–55 objective on retro modus with an exposure time of 5 ms. Post-hoc, the pictures were cut to the same size and the background was unified with FastStone Image Viewer.

Fluorescence was imaged using inverted confocal microscopes (TCS SP5, Leica, and TCS SP8, Leica) excited with a 405 nm, an argon, a 561 nm and a 633 nm laser to detect AMCA, Alexa 488, Cy3 and Alexa 647 at emission filtered wavelengths of 420–470 nm, 495–540 nm, 565–585 nm and 653–695 nm, respectively. As objective, a 10 × dry/ NA 0.40, a 20 × oil/ NA 0.75, a 40 × oil/ NA 1.25 or a 63 × oil/ NA 1.40 was used. The laser intensity and detection gain was constant for all images taken from a given section. The fluorescent images were acquired in monochrome and colour maps were applied to the images post-acquisition. Post-hoc, linear brightness and contrast adjustment were applied uniformly to the images with Fiji. Colour channels were post-hoc combined before merging the tile images using the Fiji stitching plugin^101^.

Cross-section areas of the MNTB, LSO and DNLL were determined by encircling the nuclei identified by VGluT1 labelling in Fiji with the free hand section tool. The nucleus sizes were measured from their ROIs and averaged over different sections of different labelling combinations from different animals.

## Data Availability

The datasets generated during and/or analysed during the current study are available from the corresponding author on reasonable request.

## Abbreviations

*CaBP*: Calcium binding protein
*CB*: Calbindin
*Cereb*: Cerebellum
*CN*: Cochlear nucleus
*CR*: Calretinin
*DNLL*: Dorsal nucleus of the lateral lemniscus
*GABA*: γ-Aminobutyric acid
*HCN1*: Potassium/sodium hyperpolarization-activated cyclic nucleotide-gated channel 1
*IC*: Inferior colliculus
*ICc*: Central nucleus of the inferior colliculus,
*INLL*: Intermediate nucleus of the lateral lemniscus,
*Kv1.1*: Potassium voltage-gated channel subfamily A member 1
*LL*: Lateral lemniscus
*LNTB*: Lateral nucleus of the trapezoid body
*LSO*: Lateral superior olive
*MAP2*: Microtubule-associated protein 2
*MNTB*: Medial nucleus of the trapezoid body
*MSO*: Medial superior olive
*NeuN*: Neuronal nuclear antigen
*NLL*: Nuclei of the lateral lemniscus
*PBS*: Phosphate-buffered saline
*PFA*: Paraformaldehyde
*PV*: Parvalbumin
*SOC*: Superior olivary complex,
*SPN*: Superior paraolivary nucleus
*VGAT*: Vesicular γ-aminobutyric acid transporter,
*VGluT1*: Vesicular glutamate transporter type 1
*VNLL*: Ventral nucleus of the lateral lemniscus
*VNLLvl*: Ventrolateral part of the ventral nucleus of the lateral lemniscus
